# Assembly of the Synaptonemal Complex Is a Highly Temperature-Sensitive Process That Is Supported by PGL-1 During *Caenorhabditis elegans* Meiosis

**DOI:** 10.1534/g3.112.005165

**Published:** 2013-04-01

**Authors:** Ceyda Bilgir, Carolyn R. Dombecki, Peter F. Chen, Anne M. Villeneuve, Kentaro Nabeshima

**Affiliations:** * Department of Cell and Developmental Biology, University of Michigan Medical School, Ann Arbor, Michigan 48109-2200; † Departments of Developmental Biology and Genetics, Stanford University, School of Medicine, Stanford, California 94305-5329

**Keywords:** aneuploid gametes, pachytene, synapsis, polycomplex, P-granule

## Abstract

Successful chromosome segregation during meiosis depends on the synaptonemal complex (SC), a structure that stabilizes pairing between aligned homologous chromosomes. Here we show that SC assembly is a temperature-sensitive process during *Caenorhabditis elegans* meiosis. Temperature sensitivity of SC assembly initially was revealed through identification of the germline-specific P-granule component PGL-1 as a factor promoting stable homolog pairing. Using an assay system that monitors homolog pairing *in vivo*, we showed that depletion of PGL-1 at 25° disrupts homolog pairing. Analysis of homolog pairing at other chromosomal loci in a *pgl-1*−null mutant revealed a pairing defect similar to that observed in mutants lacking SC central region components. Furthermore, loss of *pgl-1* function at temperatures ≥25° results in severe impairment in loading of SC central region component SYP-1 onto chromosomes, resulting in formation of SYP-1 aggregates. SC assembly is also temperature sensitive in wild-type worms, which exhibit similar SYP-1 loading defects and formation of SYP-1 aggregates at temperatures ≥26.5°. Temperature shift analyses suggest that assembly of the SC is temperature sensitive, but maintenance of the SC is not. We suggest that the temperature sensitive (ts) nature of SC assembly may contribute to fitness and adaptation capacity in *C. elegans* by enabling meiotic disruption in response to environmental change, thereby increasing the production of male progeny available for outcrossing.

Meiosis is the process that segregates homologous chromosomes into two separate sets so that gametes each receive a haploid genome from diploid germ cells. At the beginning of this process, the replicated chromosomes are sorted into homologous pairs through direct association between homologs. Initial homolog association is further stabilized by assembly of the synaptonemal complex (SC), a multiprotein structure (Page and Hawley 2003). In most sexually reproducing organisms, the SC is essential for establishing crossovers between homologous chromosomes by recombinational repair of meiotic double-strand breaks (DSBs). Crossovers and sister chromatid cohesion constitute a chiasma structure that physically assures bipolar attachment of the bivalent to the meiotic spindle (Watanabe 2012) and thus promotes faithful segregation of homologous chromosomes. Therefore, proper SC formation is essential for successful meiosis.

The SC exhibits a highly conserved and stereotyped tripartite structure in electron-microscopic observation. It consists of three parts: two chromosome axes and a central region structure. Axis proteins load onto the chromosomes beginning in the leptotene stage of meiotic prophase, and during the zygotene stage the axes of the two homologs start to become connected by assembly of the central region structure between them, culminating in the pachytene stage in which the central region structure links the axes along their full lengths (Zickler and Kleckner 1999). It is known that, aside from the regular tripartite structure between aligned homologous chromosomes, SC-related proteins also form aggregates, or polycomplexes, that do not associate with chromosomes. Polycomplexes can be observed before (1) synapsis; (2) during or after the dissolution of synapsis during regular meiosis of some organisms; (3) in defective meiosis of yeast mutants that are impaired in chromosome morphogenesis due to a structural or recombination defect; or (4) after treatment by colchicine or elevated non-physiological temperature in plants (reviewed in Zickler and Kleckner 1999).

In the nematode *Caenorhabditis elegans*, the SC shares a conserved tripartite structure with dimension similar to those in other organisms (Smolikov *et al.* 2008; Schild-Prufert *et al.* 2011). Cohesin proteins and other conserved chromosome axis proteins such as HIM-3 (Zetka *et al.* 1999) constitute the axial structure of the SC, whereas coiled-coil proteins such as SYP-1 (MacQueen *et al.* 2002) constitute the central region structure of the SC (reviewed in Mlynarczyk-Evans and Villeneuve 2010; Lui and Colaiacovo 2013). Pairing of homologs and assembly of SC structures are accompanied by a transient clustering of chromosomes within the nucleus and occur in a region of the gonad known as the transition zone (TZ), corresponding to the leptotene and zygotene stages of meiotic prophase. HIM-3 loads onto chromosomes in this zone (Zetka *et al.* 1999). Further, a *cis*-acting locus known as the pairing center (PC), located near one end of each chromosome, is known to strongly stabilize the initial pairing that is seen in the TZ and also to promote succeeding assembly of the SC central region (MacQueen *et al.* 2002). Assembly of the SC central region is detected by loading of SYP-1 subsequent to HIM-3 loading onto chromosomes, and it is required to stabilize the initial association of homologous chromosomes, particularly for regions of the chromosomes that are distant from the PCs (MacQueen *et al.* 2002).

Here we report that the assembly of the SC central region in *C. elegans* is a highly temperature sensitive process. Only a 1.5° increase above the standard range of culturing temperature impairs proper assembly of the SC and induces polycomplex-like aggregate formation in the wild type. This restrictive temperature becomes lower in the *pgl-1* likely-null mutant, suggesting that a P-granule component, PGL-1 (Kawasaki *et al.* 1998), confers heat resistance to the SC assembly process. We also report detailed description and validation of a use of a tool using green fluorescent protein (GFP)-LacI/*lacO* to monitor the status of homologous pairing in live animals, which facilitated the identification of PGL-1 as a factor promoting pairing and synapsis.

## Materials and Methods

### Genetics

All *C. elegans* strains were cultured following standard conditions (Brenner 1974), except that culturing temperatures were varied as indicated in the text to examine the effects of elevated culturing temperatures. The wild type strain Bristol N2 and the mutant strain *pgl-1(bn102)III* (Kawasaki *et al.* 1998) were used. Identification of *pgl-1* as a gene whose RNA interference (RNAi) knockdown causes a pairing defect was done as previously described in Dombecki *et al.* (2011). The strain AV221 carries *meIs1*, *meIs4*, and *meT8*. *meIs1* contains the coding sequence of GFP-LacI under a germline promoter, *pie-1* (Yuen *et al.* 2011). *meIs4* contains a large array of *lacO* repeat sequence and *rol-6(su1006)* gene. *meT8 III*; *IV* contains *meIs4* (see the section *Mapping of the lacO repeat array and karyotype analysis of strain AV221)*.

### Mapping of the *lacO* repeat array and karyotype analysis of strain AV221

To identify the chromosomal locus where *meIs4* (containing *lacO* array) is integrated in the AV221 strain, we first genetically mapped the locus. *meIs4* in AV221 was generated by using ionizing radiation to integrate an extra-chromosomal array constructed by coinjection of plasmid pRF4 [containing the dominant *rol-6(su1006)* marker (Mello *et al.* 1991)] and plasmid pMK19A [containing *lacO* repeats derived from pMK2A (Nabeshima *et al.* 1998)] into the genome. To map the genomic location of *meIs4*, AV221 worms (Bristol N2 strain background) were crossed with the CB4856 Hawaiian strain to generate heterozygous F1 worms. F2 progeny of these F1 heterozygotes were plated individually, and presence or absence of the Rol phenotype among their F3 progeny was used to identify F2 worms that were either homozygous for *meIs4* (homozygous Rol) or that lacked *meIs4* (homozygous non-Rol). Surprisingly, initial genotyping with the use of Snip-SNP markers (Wicks *et al.* 2001) revealed linkage of *meIs4* to markers on two chromosomes, *III* and *IV*, as indicated by cosegregation of *meIs4* with the N2 alleles of SNP markers on those chromosomes (Supporting Information, Figure S1A). Additional mapping using markers distributed along the lengths of these two chromosomes indicated complete linkage of the *meIs4* locus with most markers on chromosome *III* (except the marker at the left end) and with markers on right half of chromosome *IV* (Figure S1B). Based on this segregation pattern, we inferred that AV221 carries a reciprocal translocation between chromosomes *III* and *IV* that acts as a crossover suppressor when heterozygous, similar to several characterized translocations that serve as chromosomal balancers (*e.g.*, Herman and Kari 1989; McKim *et al.* 1988). These translocation chromosomes presumably were generated through chromosome breakage and fusion following irradiation; as we observe only a single GFP spot in pachytene nuclei of the AV221 strain, we infer that the *lacO* repeat array (*i.e.*, *meIs4*) is integrated into the crossover-suppressed region of one of these translocation chromosomes. We used a cytological approach to verify the inferred karyotype of the AV221 strain, visualizing chromosomes *III* and *IV* by chromosome painting and the *lacO* repeat array by single-locus fluorescence *in situ* hybridization (FISH). In pachytene nuclei in wild-type germlines, we saw two distinct chromosome territories painted by the two paint probes (Figure S2A, top). In the germlines of AV221, we saw a portion of the chromosome *IV* paint associated with chromosome *III* paint, and a second separate territory hybridizing only to chromosome *IV* paint (Figure S2A, bottom). We failed to detect a portion of the chromosome *III* paint associating with the second chromosome *IV* territory, which likely reflects the fact that this half-translocation contains only a small portion from the very left end of chromosome *III*. The FISH signal of the *lacO* sequence was observed to associate with the chromosome *III* paint signal. To further confirm the structure of the translocation chromosome that carries *lacO* repeats, we applied a paint probe for chromosome *III* that lacks the left end part (about 1Mb long) in combination with a paint probe for the left half of chromosome *IV*. These two paint signals are associated with distant territories in the wild type (Figure S2B, top), but they are consistently near each other in AV221 (Figure S2B, bottom), confirming that these two chromosome segments are fused in AV221. Based on the combined genetic and cytological data, we conclude that AV221 carries a reciprocal translocation between chromosomes *III* and *IV* with the structure diagrammed in Figure S2C and that the *lacO* array is integrated into the half translocation chromosome that consists of most of chromosome *III* (lacking the left end) linked to the left half of chromosome *IV*. We named this translocation *meT8*.

### Cytological analysis

Gonad dissection, fixation for DAPI staining, IF, FISH, chromosome paint, and imaging using the DeltaVision deconvolution microscopy system or Olympus BX61 were conducted basically as previously reported (Nabeshima *et al.* 2004; 2005; 2011). Worms were picked at the late L4 stage and cultured for 24 hr (if not specified in the figure legend) at indicated temperatures before dissection. The following primary antibodies were used at the indicated dilutions: rabbit anti-HIM-3 (Zetka *et al.* 1999), 1:200; rabbit anti-HIM-3 generated in this study using peptide ATKEQIVEHRESEIPIASQWKATFPVD(GC), 1:1000; guinea pig anti-SYP-1 (MacQueen *et al.* 2002), 1:200; guinea pig anti-SYP-1 generated in this study using peptide DNFTIWVDAPTEALIETPVDDQS(GC), 1:1000; and rabbit anti-RAD-51 (Colaiacovo *et al.* 2003), 1:200. The experiments at nonconventional culture temperatures (*i.e.*, other than 20 and 25°) were done using the Incufridge (Revolutionary Science, Shafer, MN) whose temperature was adjusted and confirmed using at least three thermometers. The temperature of the low-temp incubator model 815 (Thermo Fisher Scientific, Waltham, MA) for 20° and 25° were also adjusted in the same manner.

### Scoring of homologous pairing

Three gonads that spanned the distal end to pachytene exit were imaged in multiple frames for N2 and *pgl-1*(*bn102*) cultured at 26° for 24 hr after L4 stage. All nuclei that were clearly resolved from other nuclei were examined as previously described in Dombecki *et al.* (2011). One N2 gonad showed partial decrease in pairing frequency as shown in Figure S3. The data from this gonad were not combined with the data set from the other two N2 gonads. Fisher’s exact test (two-tailed) was used for statistical analysis to compare the pairing efficiency in the same subzone between *pgl-1(bn102)* and wild-type worms. For ZIM-2 pairing assay, ZIM-2 IF signal was treated in the same way as the FISH signals for scoring.

### Quantification of RAD-51 foci

The dissected gonads stained by SYP-1 and RAD-51 IF, which covered the distal end to the pachytene exit, were imaged in multiple frames for N2 and *pgl-1*(*bn102*). For *pgl-1*(*bn102*), a gonad that exhibited SYP-1 aggregate formation in the most of the pachytene stage was chosen for analysis. All nuclei that were clearly resolved from other nuclei were examined as previously described in Dombecki *et al.* (2011).

### Scoring of SYP-1 aggregate formation

The gonads that were stained by HIM-3 and SYP-1 immunostaining and DAPI were visually examined with an Olympus BX61 microscope. The region of each gonad with HIM-3 staining on chromosomes was defined as the meiotic region. This region was subdivided into three zones with equal length. Gonads exhibiting more than five nuclei with SYP-1 aggregates present per zone in either of the two later zones were scored as positive for SYP-1 aggregate formation. If SYP-1 aggregate formation was observed only in the first zone, the gonad was scored as positive for SYP-1 aggregate formation only when more than five nuclei with SYP-1 aggregates were observed toward the proximal end of this zone. Gonads in which SYP-1 aggregates were present only in the beginning (distal portion) of this zone were not scored as positive for SYP-1 aggregate formation as SYP-1 aggregates are sometimes observed in this region of normal gonads just prior to SC assembly.

## Results

### Live observation of homologous pairing status in *C. elegans* germline using the GFP-LacI/*lacO* system

**Figure 1:**
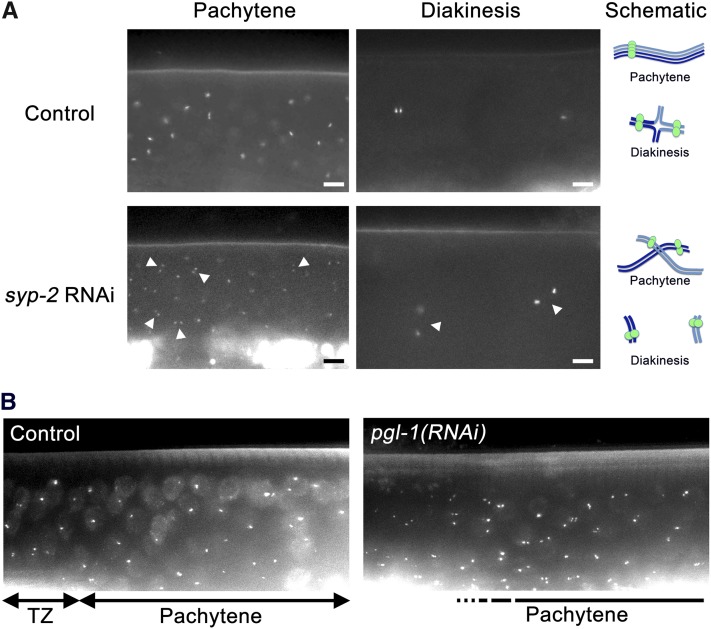
Fluorescent signals in live animals carrying a GFP-LacI/*lacO* system report the status of meiotic homologous pairing. (A) Shown are the pachytene (left) and diakinesis (right) regions of a gonad in live AV221 worms without any treatment (top) and after *syp-2*(*RNAi*) by injection of double-stranded RNA targeted to the *syp-2* gene into their parental worms (bottom). The locations of nuclei with unpaired signals in the *syp-2*(*RNAi*) gonad are indicated by arrowheads. In some cases, the nuclei can be identified by diffuse GFP signals. The bright diffuse fluorescent signals seen in the lower part of a panel are the autofluorescence of the gut, whereas the fluorescent dots in the upper part of a panel are the signals corresponding to LacI-GFP. Worms were cultured at 20°. Bar: 5 µm. Schematics depicting the inferred configuration of chromosomes are shown at the right. (B) The TZ and the pachytene region of a gonad in live AV221 worms fed either bacteria carrying the empty RNAi vector (left) or an RNAi plasmid expressing double-stranded RNA targeted to *pgl-1* gene (right). Note that many nuclei are visualized by bright diffuse GFP signals. TZ and the pachytene regions are defined by nuclear morphology. The beginning of the pachytene zone is not clear for *pgl-1*(*RNAi*) due to the lack of a clear TZ, which is indicated by a dashed line. Worms were cultured at 20° until L4 stage, then shifted to 25° for 24 hr.

As a means to directly examine the status of meiotic homologous pairing *in vivo*, we built a strain, AV221, that carries a transgene insertion (*meIs1*) expressing GFP-LacI using the germline-specific *pie-1* promoter (Praitis *et al.* 2001; Yuen *et al.* 2011) and a large array of *lacO* repeats integrated into a single genomic locus on chromosome *III* (*meIs4*). In this strain, the event that integrated the *lacO* array onto chromosome *III* also generated a reciprocal translocation (*meT8*) between chromosomes *III* and *IV* (see the section *Materials and Methods*, Figure S1, and Figure S2). Because this modified karyotype does not interfere with meiosis (progeny viability and frequency of male production for worms homozygous for *meT8* are comparable with the wild type), we used the AV221 strain to visualize homologous chromosome pairing during meiotic prophase in the germlines of live worms. AV221 worms exhibit GFP signals in meiotic prophase nuclei in the gonads of live worms (Figure 1), beginning in the TZ, where germ cells enter meiotic prophase and meiotic pairing begins. We observed two distinct GFP dots representing unpaired homologs in some nuclei in TZ (Figure 1B; TZ), and one GFP dot (or two closely associated dots) in basically all nuclei in the middle region of a gonad that corresponds to the pachytene stage (Figure 1, A and B; controls), which represent paired homologous loci. In diakinesis-stage oocytes we observed two discrete but closely located dots in the wild type background, which reflects the configuration of the chromosomes as a bivalent in which the homologs are connected by a chiasma (Figure 1A; control). Furthermore, when SC formation was impaired by RNAi for an SC central region component, SYP-2 (Colaiacovo *et al.* 2003), we observed two well-separated GFP signals in nuclei in both the pachytene and diakinesis stages (Figure 1A; *syp-2* RNAi, arrows). Together these results validate the use of the AV221 strain to examine meiotic pairing status in live animals.

### RNAi against *pgl-1* gene impairs meiotic homologous pairing

We conducted a screen for genes promoting pairing and synapsis with the AV221 strain by individually knocking down 1762 preselected genes through RNAi. This screen was performed at 25° to increase the intensity of GFP signal. The preselected genes included genes exhibiting germline-enriched expression (Reinke *et al.* 2000, 2004) and genes that share expression patterns with known genes required for meiotic homologous pairing (Kim *et al.* 2001). Using feeding RNAi clones from a library of *C. elegans* coding sequences (Fraser *et al.* 2000), we found a set of clones that significantly increased the frequency of unpaired GFP signals in the pachytene region of the gonad. This set included the RNAi clone against the *pgl-1* gene [Figure 1B, *pgl-1*(*RNAi*)], which suggests that loss-of-function of *pgl-1* gene impairs proper homologous pairing at 25° during *C. elegans* meiosis.

### *pgl-1*(*bn102*) mutant exhibits a defect in homologous pairing reminiscent of SC-defective mutants

**Figure 2:**
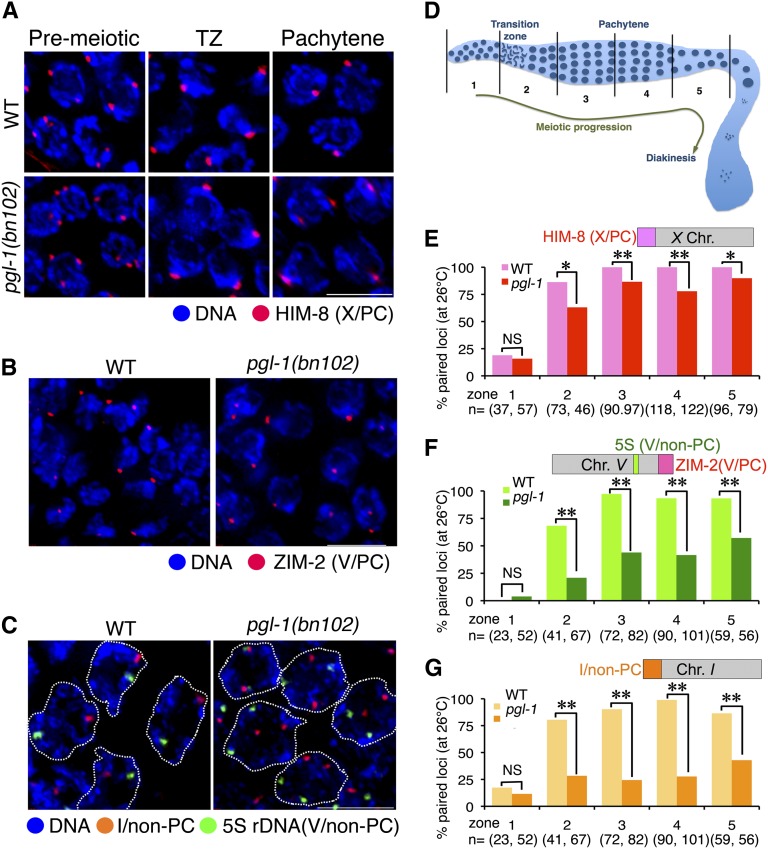
The *pgl-1* mutant exhibits a decrease in meiotic homologous pairing. (A) IF staining of HIM-8 (marking the PCs, of the *X* chromosomes, red) with DNA counter staining (blue) in whole mount gonads from wild-type (top) and *pgl-1*(*bn102*) mutant (bottom) worms cultured at 26° for 24 hr. Bar: 5 µm. (B) IF staining of ZIM-2 (marking the PC of chromosome *V* and appearing in the TZ, red) in the wild type (left) and the *pgl-1*(*bn102*) mutant (right) with DNA counter staining (blue) cultured at 26°. Bar: 5 µm. (C) FISH visualizing the non-PC end of chromosome *I* (red) and 5S rDNA locus on the chromosome *V* (green) with DNA counter staining (blue) in pachytene nuclei (outlined with dashed lines) of wild-type (left) and the *pgl-1*(*bn102*) mutant (right) worms cultured at 26°. Bar: 5 µm. (D) Schematic diagram of zones (1−5) of the gonad used for scoring of pairing efficiency. The distal end is shown toward the left. (E, F, G) The pairing efficiency at the indicated locus is presented as the percentage of nuclei in each zone with paired signals. Asterisks indicate a statistically significant difference between wild type and *pgl-1* in the corresponding stage (*0.001 ≤ *P* < 0.01, **P < 0.001). NS indicates not statistically significant.

To further characterize the effect of loss of *pgl-1* function in meiotic homologous pairing, we examined the meiotic phenotype of a likely-null allele, *pgl-1*(*bn102*), which has a premature stop codon at Trp-329 and does not produce any truncated PGL-1 protein that is recognized by antibodies raised against a peptide spanning from Asn-195 to Val-551 (Kawasaki *et al.* 1998). It was reported that *pgl-1(bn102)* exhibits a sterile phenotype in a temperature-dependent manner and becomes completely sterile at 26°, which indicates that *pgl-1*(*bn102*) is fully defective in reproduction at 26°. At 25°, the temperature at which our RNAi screen was conducted, the reported sterility of *pgl-1*(*bn102*) is 75–85%. Therefore, we examined homologous pairing activity in *pgl-1*(*bn102*) at 26°. In the *pgl-1(bn102)* mutant, nuclei with chromosome clustering, which is a hallmark of leptotene/zygotene stages, were not confined to a small subregion of the gonad, known as TZ in the wild type. Instead, we observed a mixture of nuclei with tightly clustered chromosomes and nuclei with less tightly clustered or unclustered chromosomes after meiotic entry (defined by the emergence of nuclei with clustered chromosomes), making a distinction between the TZ and pachytene stage difficult. Thus, we simply subdivided meiotic prophase region to four equal-length subregions for scoring. First, we examined the status of homologous pairing of the PC locus of the *X* chromosome by using indirect immunofluorescence (IF) staining of HIM-8, which localizes mainly on this locus (Phillips *et al.* 2005). We scored the frequency of nuclei with paired HIM-8 signals (Figure 2A) in five zones that correspond to the premeiotic stage and four substages of meiotic prophase (Figure 2D). In the premeiotic zone (zone 1), the frequency of nuclei with paired signals is less than 20% in both wild-type and *pgl-1* mutant. As shown in Figure 2E, in both the wild-type and *pgl-1* early meiotic prophase (mainly the leptotene/zygotene stage, zone 2), pairing frequency increased to 60–80%. In later stages (zones 3–5), pairing frequency increased to and persisted at 100% in wild type, whereas it remained at ~80% in the *pgl-1* mutant. The fact that meiotic homologous pairing at the *X*-PC is only moderately impaired indicates that the synapsis-independent pairing activity of the *X*-PC is retained in the *pgl-1(bn102)* mutant. We also examined pairing at the PC of chromosome *V* by immunostaining for ZIM-2, the PC-binding protein specific to chromosome *V* (Phillips and Dernburg 2006). ZIM-2 foci are present only in the nuclei of early meiotic prophase, which limits our scoring of pairing at the *V*-PC locus to only this stage. In most nuclei exhibiting ZIM-2 staining, ZIM-2 was observed as a single focus or two closely associated foci both in wild type and the *pgl-1(bn102)* mutant (Figure 2B). The frequency of nuclei with paired ZIM-2 signal is 91% in the wild type (n = 35) and 79% in the *pgl-1(bn102)* mutant (n = 72). Their difference is not statistically significant (two-tailed Fisher’s exact test, *P* = 0.17). This result indicates that *V*-PC is also capable of pairing in the *pgl-1(bn102)* mutant, at least in early meiotic prophase. We next examined pairing activity in non-PC loci in *pgl-1* mutants cultured at 26°. Specifically, we examined homologous pairing at the 5S rDNA locus on chromosome *V* and at the non-PC end of chromosome *I* by using FISH (Figure 2C). As shown in Figure 2, F and G, both loci showed an increased frequency of paired FISH signals in zone 2 (relative to zone 1) in the *pgl-1* mutant, but the pairing frequency was significantly lower compared with the wild type. Moreover, homologous pairing frequencies in the *pgl-1* mutant remained lower (20–60%) in zones 3–5, where pairing reached 85–100% in the wild type. Taken together, we conclude that pairing is impaired moderately at the PC and severely at the non-PC loci in the *pgl-1* mutant. This pairing profile is reminiscent of mutants such as *syp-1* (MacQueen *et al.* 2002) and *syp-2* (Colaiacovo *et al.* 2003) that lack components of the SC central region and thus cannot assemble the SC.

In general, wild-type worms that were cultured at 26° exhibited successful meiotic homologous pairing. However, we found one example of a gonad that showed lower pairing efficiency in the middle to late pachytene stage (Figure S3), which suggests that culturing wild-type *C. elegans* at 26° may occasionally affect meiotic homologous pairing (see the section “*Wild-type gonads exhibits pgl-1-like SYP-1 aggregate formation at temperatures ≥26.5°*”).

### *pgl-1*(*bn102*) genetic mutant exhibits aggregates of an SC central region component

**Figure 3:**
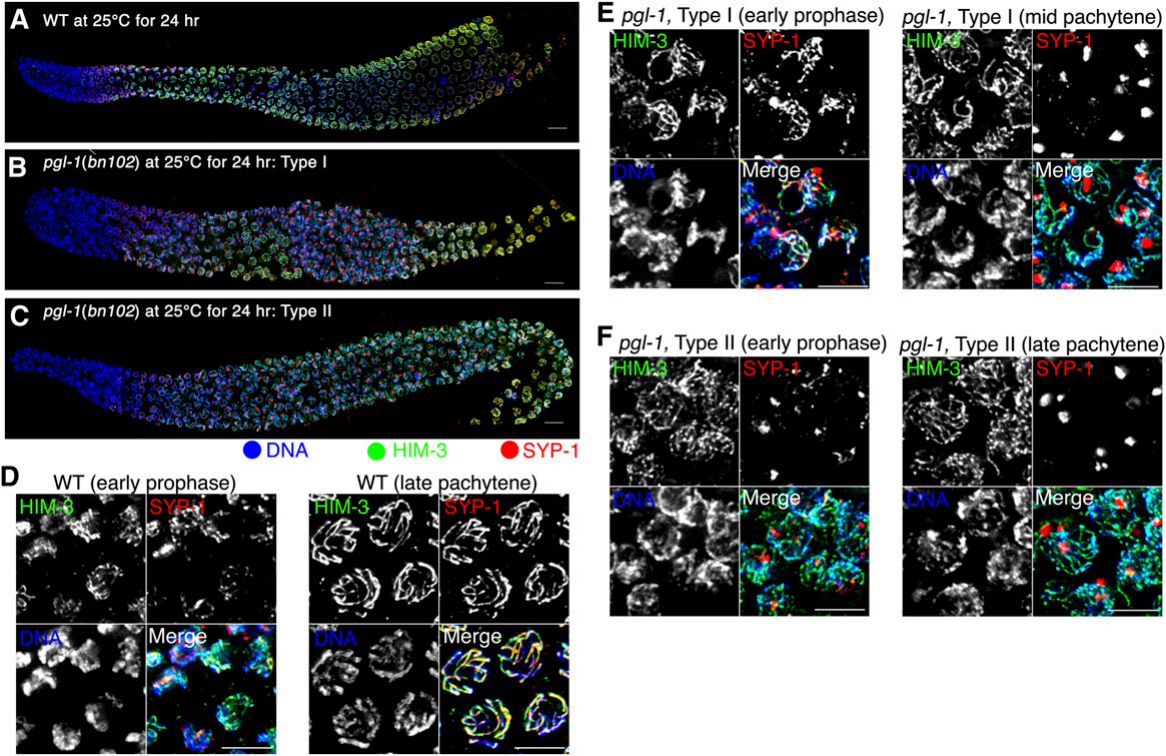
The *pgl-1* mutant exhibits SYP-1 aggregate formation. (A−C) IF staining of HIM-3 (green) and SYP-1 (red) in a wild-type (A) and a *pgl-1*(*bn102*) mutant (B and C) whole mount gonad cultured at 25° for 24 hr. In the Type I gonad (B), SYP-1 aggregates are present in the middle-late pachytene regions of the gonad but are not present in early meiotic prophase; in the Type II gonad (C), SYP-1 aggregates are detected throughout the meiotic region. The distal end is toward the left. Bar is 10 µm. (D−F) Closeup of panels (A), (B), and (C), respectively, in early meiotic prophase (left) and the middle or late pachytene region (right). Bar: 5 µm.

The similarity in pairing defect to the SC-defective mutants suggests that SC assembly fails in the *pgl-1(bn102)* mutant at 25°. Therefore, we examined SC formation by visualizing the SC central region and chromosomal axis using IF staining for SYP-1 and HIM-3 (Figure 3). For this experiment, worms were cultured at 25° because homologous pairing occasionally fails at 26° in the wild-type control as described previously, and loss of function of *pgl-1* still leads to a pairing defect at 25°, as shown in Figure 1. As expected from their meiosis-specific expression, SYP-1 and HIM-3 proteins appear after meiotic entry in the *pgl-1* mutant, similarly to the wild type (Figure 3, A−C). In the wild type, HIM-3 loads onto the chromosomal axes before SYP-1 loading on the interface between the axes of two aligned homologs (Figure 3D). After SYP-1 loading, HIM-3 and SYP-1 staining become overlapped and track synapsed chromosomes, demonstrating completion of SC assembly. In contrast, SYP-1 was not detected on the chromosomes in many meiotic prophase nuclei in the *pgl-1* mutant, and instead formed aggregates. These aggregates were observed in most middle prophase and many late prophase nuclei (but not in early prophase nuclei) in 56% of germlines examined (Type I; Figure 3, B and E). In 4% of gonads, SYP-1 aggregates were also observed from early to late meiotic prophase (Type II; Figure 3, C and F). There were also other types of gonads exhibiting SYP-1 aggregates (see Figure S4). The mean number of SYP-1 aggregates seen in midprophase nuclei was 1.24 ± SD 0.55 (n = 50). In these nuclei with SYP-1 aggregate(s), no SYP-1 was loaded onto the chromosomes (n = 50). In contrast, HIM-3 was observed as stretches along chromosomes in these nuclei. The formation of SYP-1 aggregates, together with the observed pairing profiles, strongly suggests that the pairing defect in *pgl-1* is caused by a defect in proper SC assembly, which is necessary to stabilize initial pairing.

### Accumulation of recombination intermediates in the *pgl-1* mutant

**Figure 4:**
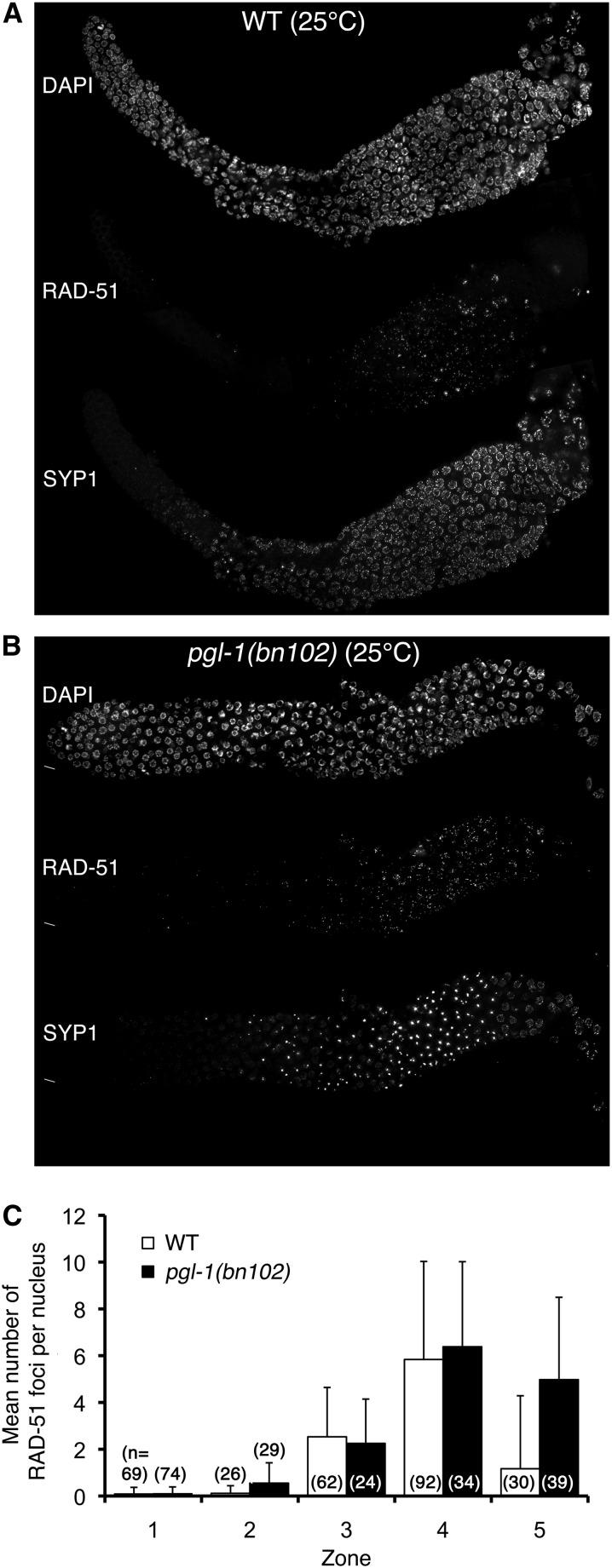
Accumulation of RAD-51 foci in *pgl-1*(*bn102*) worms cultured at 25°. (A and B) IF staining of RAD-51 (middle) and SYP-1 (bottom) with DNA counterstaining (top) in the wild-type (A) and the *pgl-1(bn102)* mutant (B) whole mount gonad cultured at 25° for 24 hr after L4 stage. (C) Average number of RAD-51 foci per nucleus in five zones of the gonad. Zones are defined as in Figure 2D. Error bar indicates SD.

Because proper assembly of the SC is necessary to complete DSB repair by crossover formation (Colaiacovo *et al.* 2003), we tested whether meiotic DSB repair might be defective in the *pgl-1* mutant when SYP-1 is present in aggregates rather than assembled into SC. Specifically, we monitored SYP-1 aggregation by IF for SYP-1 and meiotic DSB formation and repair by IF for RAD-51, which marks intermediates of DSB repair as foci on chromosomes. As shown in Figure 4, A and B, an increase of RAD-51 foci indicative of meiotic DSB formation was observed in the early-middle pachytene stage in both wild-type and *pgl-1* mutant worms cultured at 25° for 24 hr. In late pachytene stage, however, RAD-51 foci persisted in *pgl-1* mutants that exhibited SYP-1 aggregates, whereas they disappeared in wild-type worms that did not show SYP-1 aggregates. After pachytene exit, RAD-51 foci disappeared, indicating that DSBs were eventually repaired, presumably by homologous recombination between sister chromatids or by nonhomologous end-joining. Quantification of the number of RAD-51 foci clearly demonstrates that RAD-51 foci are significantly (*P* < 0.0001; Mann-Whitney two-tailed test) more abundant in the *pgl-1* mutant compared with the wild type after the middle-late pachytene stage (zone 5, Figure 4C). This result indicates that *pgl-1* mutants cultured at 25° are defective in timely repair of meiotic DSBs, most likely due to the fact that SYP-1 is present in aggregates rather than on chromosomes.

### Wild-type gonads exhibits *pgl-1*-like SYP-1 aggregate formation at temperatures ≥26.5°

**Figure 5:**
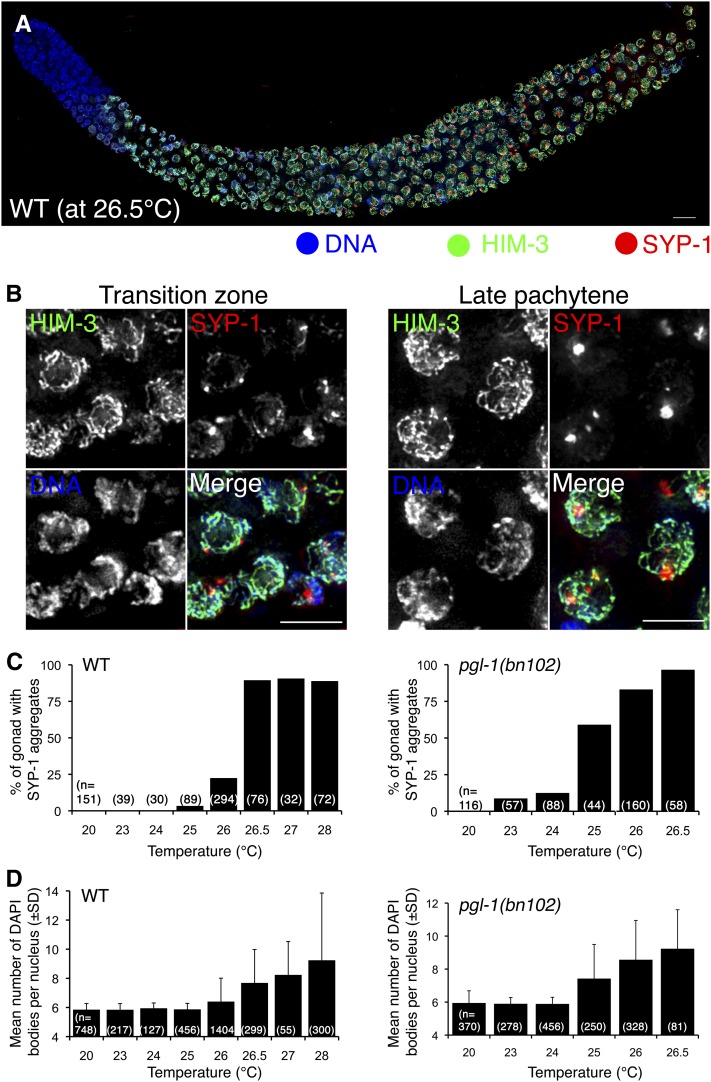
The wild type exhibits SYP-1 aggregate formation similar to the *pgl-1* mutant at high temperatures, which correlates with a failure in chiasma formation. (A) IF staining of HIM-3 (green) and SYP-1 (red) in the wild-type whole mount gonad cultured at 26.5° for 24 hr. This is a representative wild-type gonad, which exhibits the Type II pattern in which SYP-1 aggregates are detected both in the early and middle-late pachytene regions. The distal end is toward left. Bar: 10 µm. (B) Closeup of the (A) in the TZ (left) and the late pachytene region (right). Bar: 5 µm. (C) the frequency of the gonads exhibiting SYP-1 aggregate formation at the indicated temperature in the wild type (left) and the *pgl-1(bn102)* mutant (right). (D) Average number of DAPI-stained bodies in a diakinesis nucleus in the wild type and the *pgl-1(bn102)* mutant cultured at the indicated temperatures for 48 hr after L4 stage. Error bar indicates SD. Prior to L4 stage, worms were cultured at 20°.

We noticed that at 25°, some nuclei in a small fraction of the wild-type gonads (3 of 89) exhibited SYP-1 aggregates whose appearance was similar to those in the *pgl-1* mutant. Because SYP-1 aggregation is not observed at 20°, we suspected that culturing worms at a temperature greater than 20° induces the SYP-1 aggregation phenotype. We examined this possibility by culturing wild-type worms at various temperatures ≥20° for 24 hr. At 26.5°, 43% of the wild-type gonads examined exhibited SYP-1 aggregates throughout the gonad (Type II; n = 55; Figure 5, A and B). As in the *pgl-1* mutant, there were other types of gonads exhibiting SYP-1 aggregates (Figure S4), and SYP-1 aggregates disappeared after the pachytene exit (Figure S5). As shown in Figure 5C, SYP-1 aggregates were very infrequent in worms cultured at temperatures ≤ 25°. Wild-type worms cultured at 26° showed a moderate increase in the fraction of worms with SYP-1 aggregates, which is consistent with the occasional pairing failure we had observed at this temperature. At 26.5, 27, or 28°, the fraction of worms with SYP-1 aggregates in their gonads reached almost 100%. Therefore, we conclude that culturing worms at temperatures ≥26.5° causes considerable SYP-1 aggregate formation in the wild type. In the *pgl-1* mutant, SYP-1 aggregate formation occurs at a lower temperature, 25°, which suggests that PGL-1 suppresses SYP-1 aggregate formation at temperatures below 26.5° in the wild type (Figure 5C, left). We examined this possibility by determining the temperature dependency of SYP-1 aggregate formation in the *pgl-1* mutants. At 20°, *pgl-1* mutants did not show SYP-1 aggregation and SYP-1 was loaded on the chromosomes, but at 23° and 24° we observed an increase of SYP-1 aggregation in *pgl-1* mutants. SYP-1 aggregation was seen in 59% and 83% of *pgl-1* worms at 25° and 26° respectively (Figure 5C, right). Therefore we conclude that PGL-1 confers heat tolerance to SC central region assembly to prevent aggregate formation at these temperatures.

### Failure in bivalent formation at high temperatures correlates with SYP-1 aggregate formation

A defect in SC assembly and succeeding failure in crossover formation could lead to failure in bivalent formation in the diakinesis stage. We examined this possibility by counting the number of 4’,6-diamidino-2-phenylindole (*i.e.*, DAPI)-stained bodies in diakinesis oocytes. We found that failure in bivalent formation indeed occurs at high temperatures and the temperature-dependence of this phenotype correlates well with SYP-1 aggregate formation. As shown in Figure 5D (left), at temperatures lower than 26°, the average number of DAPI-stained bodies in the wild type was approximately 6, indicating that bivalent formation is successful among all six chromosomes. At temperatures ≥26°, we observed diakinesis nuclei exhibiting >6 DAPI-stained bodies (but ≤12), and the average number of DAPI bodies was larger than 6, clearly indicating impairment of bivalent formation at these temperatures in the wild type. The *pgl-1(bn102)* mutant exhibited failure in bivalent formation at lower temperatures compared with the wild type (Figure 5D, right). Considering the essential function of SYP-1 in crossover formation (MacQueen *et al.* 2002), these results strongly support the possibility that impaired SYP-1 loading is the cause of a defect in crossover formation and consequent failure in bivalent formation in both the wild type and *pgl-1* mutants at temperatures ≥26.5° and 25°, respectively.

### High-temperature treatment impairs the assembly step of SC formation both in the wild type and the *pgl-1* mutant

**Figure 6:**
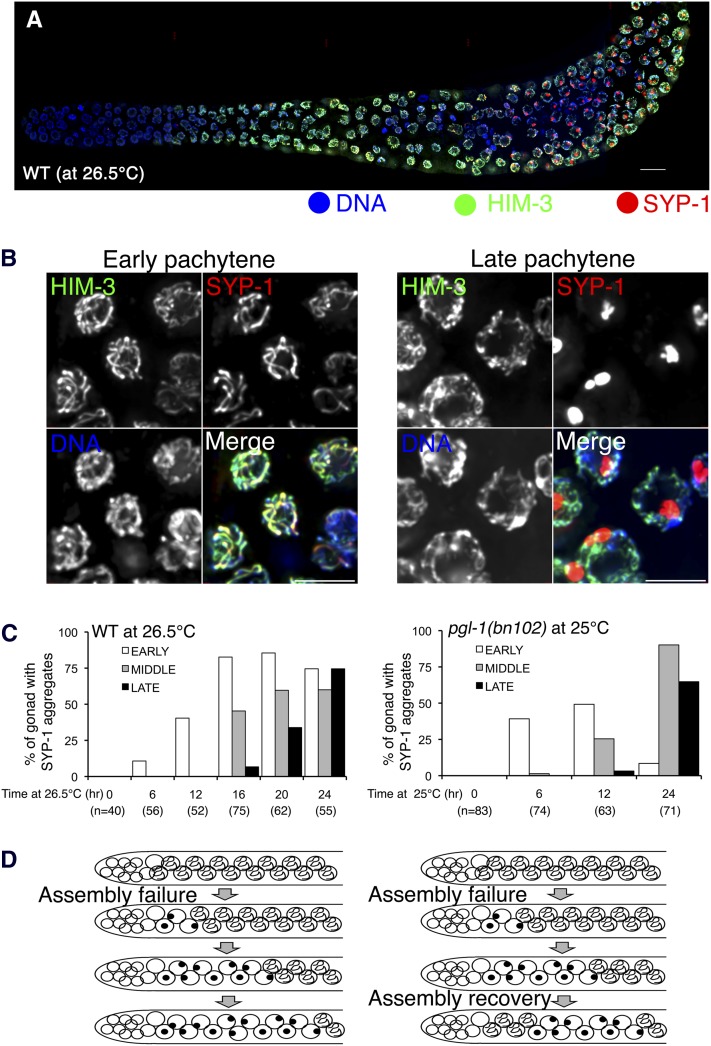
SYP-1 aggregates first appear in the early pachytene stage in both the wild type and the *plg-1* mutant cultured at high temperatures. (A) IF staining of HIM-3 (green) and SYP-1(red) in the wild-type whole mount gonad cultured at 26.5°. This example is a Type I gonad, with assembled SCs in the early pachytene region and aggregates in the middle-late pachytene region. (B) Closeup of (A) in the early pachytene stage (left) and the late pachytene region (right). Bar: 5 µm. (C) Graphs showing the frequencies of gonads exhibiting SYP-1 aggregate formation in the early (white), middle (gray), or late pachytene regions (black) when the worms were cultured for the indicated amount of time at elevated temperature (26.5° for the wild type; 25° for the *pgl-1(bn102)* mutant). (D) Schematic depicting the inferred progression through meiotic prophase of nuclei that failed in SC assembly, either without (left) or with recovery of the ability to assemble SC in nuclei entering meiotic prophase at later time points after the temperature shift.

The presence of SYP-1 aggregates in early meiotic prophase nuclei (*e.g.*, in Type II gonads) suggests that SC assembly fails. On the other hand, the presence of gonads in which SYP-1 aggregates are observed only in the middle-late pachytene stage of prophase (*e.g.*, Type I gonads) suggests two possibilities: (1) SC assembly is intact, but its maintenance fails and the SC prematurely disassembles, producing SYP-1 aggregates, or (2) SC assembly fails upon a shift up to nonpermissive temperature, but recovery from the failure occurs later, which restores proper SC assembly. In the latter possibility, germ cells that had failed in SC assembly in the TZ (before the hypothesized recovery) would be displaced toward the proximal end of the gonad by the continuous movement of nuclei from the distal to the proximal end of the gonad; germ cells entering the TZ after recovery would then be competent to assemble the SC, producing the observed Type I pattern in which nuclei with assembled SCs are observed distal to nuclei with SYP-1 aggregates. Although the Type I pattern predominated in the *pgl-1* mutant (56% of gonads at 25°), the Type I pattern was also seen in some wild-type gonads cultured at high temperatures for 24 hr, albeit much less frequently (11% of gonads; Figure S4). Figure 6, A and B shows such an example from a wild-type worm cultured at 26.5°. Thus, to address whether assembly or maintenance of the SC (or both) is defective at greater temperatures, we conducted a time-course experiment.

The continuous movement of germ-cell nuclei from the distal end toward to the proximal end is supported by proliferation of germline stem cells and premeiotic germ cells (Kadyk and Kimble 1998; Maciejowski *et al.* 2006), and it is estimated that it takes 12−18 hr to reach to the middle pachytene stage from the leptotene/zygotene stage at 20° (Jaramillo-Lambert *et al.* 2007). Thus, if the primary defect is in SC assembly, we should observe SYP-1 aggregates only in early meiotic prophase (*i.e.*, early pachytene) as the earliest phenotype after the temperature shift up. If a maintenance defect (*i.e.*, premature SC disassembly) occurs, then we should observe SYP-1 aggregates in the middle-late pachytene stage as one of the earliest phenotypes observed, even within 12 hr after temperature shift up. We picked worms in the L4 larval stage and cultured them for 24 hr before worms were fixed and stained with SYP-1 and HIM-3 IF. This 24-hr culturing period was divided into two parts: the first part was carried out at 20°. Then at the beginning of the second part, the temperature was shifted up to 26.5° or 25° for the wild type or *pgl-1* mutant, respectively. When we cultured worms at the greater temperatures for only 6 hr, SYP-1 aggregates were observed only in the early pachytene stage both in the wild type (Figure 6C, left) and the *pgl-1(bn102)* mutant (Figure 6C, right). When we cultured worms for a longer time at the greater temperatures (16 and 12 hr for wild type and *pgl-1* mutants, respectively), we started to observe SYP-1 aggregates in the middle pachytene in addition to the early pachytene. SYP-1 aggregates were observed in a considerable number of the late pachytene nuclei only when we cultured worms at the greater temperatures for ≥ 20 hr. This result strongly suggests that SYP-1 aggregate formation both in the wild type and *pgl-1* mutants primarily reflects a failure in SC assembly. In addition, this defect appears to be transient, particularly in the *pgl-1* mutant, since the frequency of detecting SYP-1 aggregates in the early pachytene stage decreased as the worms spent more time at the elevated temperature, presumably due to the recovery of the SC assembly activity (schematically shown in Figure 6D, right).

## Discussion

In this study, we demonstrate that SC assembly in *C. elegans* is a temperature-sensitive process. The standard range of laboratory culture temperatures for *C. elegans* is 15−25° (Brenner 1974), and we found that an increase of only 1.5° above this range causes the SC central region proteins to form aggregates instead of loading onto chromosomes, indicating that SC assembly is exquisitely sensitive to temperature in this organism. A temperature-sensitive nature of the SC assembly may be widely conserved among sexually reproducing organisms, as Loidl previously reported that *Allium ursinum*, a plant that is normally cultured at 5−15°, exhibited leptotene arrest, SC component aggregate formation, and aberrant SC formation when cultured at 35° (Loidl 1989).

Aggregate formation of SC components in *C. elegans* has been previously observed under the following conditions: (1) when axis formation is impaired in meiotic mutants such as *him-3* (Couteau *et al.* 2004), *htp-3* (Goodyer *et al.* 2008), or *rec-8coh-3coh-4* (Severson *et al.* 2009); (2) when SC proteins are expressed precociously in premeiotic germ cells (Merritt and Seydoux 2010; Voronina *et al.* 2012); (3) when axis formation is intact but regulation of SC assembly is impaired (Smolikov *et al.* 2008); (4) in the dynein mutant *dhc-1*(*or195*); *dlc-1 RNAi* at 25°, which is proposed to be defective for licensing of SC formation (Sato *et al.* 2009); and (5) when the SYP-1 protein is expressed outside of *C. elegans* meiosis (*i.e.*, in *Xenopus* cells) (Schild-Prufert *et al.* 2011). Because the axis protein HIM-3 was loaded onto chromosomes and did not localize to aggregates, axis formation appears to be intact in both the wild type and *pgl-1* mutants at the high temperatures. These data, together with our time-course experiments, strongly suggest that the high-temperature SC defects in both the wild type and the *pgl-1* mutant primarily reflect an impairment in assembly of the central region of the SC. Although we cannot exclude the possibility that maintenance of assembled SC might also be partially impaired at high temperatures, our time-course data do indicate that previously assembled SC is significantly more heat resistant than assembling SC.

How does PGL-1 support proper SC assembly at moderately high temperatures (*i.e.*, 22−25°)? It is possible that multiple mechanisms may contribute to this activity. For example, it was recently reported that PGL-1 inhibits premature expression of several meiotic gene products at 20° by promoting the silencing activity of FBF-2, a member of the PUF family of RNA-binding proteins. Further, failure in this mechanism causes aggregate formation of SC components in the absence of another PUF family RNA-binding protein, FBF-1 (Voronina *et al.* 2012). It is possible that PGL-1 supports proper SC assembly at greater temperatures through a similar mechanism, *e.g.*, PGL-1 might help to maintain the right balance among the SC subunits and/or among other proteins, which might be required for proper SC assembly. However, the phenotype of *pgl-1* at high temperatures is distinct from that of the *pgl-1*; *fbf-1* double mutant at 20° (Voronina *et al.* 2012) in several ways, so it is unlikely that PGL-1 supports SC assembly solely by buffering against heat-labile FBF-1 function. Another possibility is suggested by a recent proteomics analysis (Tohsato *et al.* 2012) showing that the relative levels of several heat shock proteins were altered in the *pgl-1* mutant cultured at 25° compared with wild-type controls. Given the tendency of SC central region proteins to aggregate, it is easy to envision that altered levels of heat shock proteins might indirectly affect the ability to properly assemble the SC at high temperatures. Furthermore, it is also known that P-granules, which contain PGL-1, associate with the nuclear pore complex, NPC (Schisa *et al.* 2001). Considering the fact that depletion of NPC component NPP-22 suppresses the embryonic lethal phenotype of the *dhc-1*(*or195ts*) mutant (O’Rourke *et al.* 2007) and that *dhc-1*(*or195ts*); *dlc-1 RNAi* germlines exhibit SYP-1 aggregate formation at 25° (Sato *et al.* 2009), one can speculate that PGL-1 might affect SC formation by modifying dynein activity, either directly or through the function of NPC.

Does a temperature-sensitive nature of the SC assembly provide any potential benefit to *C. elegans*? The answer seems to be yes. As described already, *C. elegans* exhibits an exquisite temperature sensitivity of the SC, and increasing the sensitivity might have been evolutionarily advantageous for *C. elegans*. It is known that *C. elegans* progressively loses fecundity at temperatures greater than 24° (Hirsh *et al.* 1976), most likely due to production of nonfunctional sperm (Harvey and Viney 2007), and becomes completely sterile above 27° (Felix and Braendle 2010). The defect in SC assembly that we report here occurs at a slightly lower temperature (26.5°); therefore, temperature- sensitive SC assembly would lead to production of aneuploid embryos at the intermediate temperatures that do not render the worms completely sterile. Furthermore, if a temperature shift occurs after the successful production of functional sperm, aneuploid embryos might be produced even at temperatures greater than 27°. This idea that increased temperature can lead to chromosome missegregation is supported by the well-known fact that a short heat shock treatment of worms (at 30° for 6 hr) is commonly used to generate males from hermaphrodites (Hodgkin 1999). Generation of *XO* males from *XX* hermaphrodites of *C. elegans* at high temperatures suggests that nondisjunction of the *X* chromosome is induced by heat treatment, which is consistent with our finding that SC assembly and bivalent formation are impaired at temperatures ≥26.5°. Although the population of males is very low at the standard culturing temperatures (Hodgkin 1983) and hermaphrodites usually reproduce by self fertilization, it was experimentally demonstrated that outcrossing through the male is advantageous in maintaining fitness and promoting adaptation in *C. elegans* (Morran *et al.* 2009). Thus, we speculate that the highly temperature-sensitive nature of SC assembly in *C. elegans* may have evolved as a part of a protective mechanism that promptly responds to a potentially harmful change in thermal environment, increasing the fitness or adaptation capacity via male production.

## Supplementary Material

Supporting Information
