# Supplementary material for: Assembly of the Synaptonemal Complex Is a Highly Temperature-Sensitive Process That Is Supported by PGL-1 During Caenorhabditis elegans Meiosis
Source: G3 (Bethesda). 2013 Apr 1;3(4):585–95. doi: 10.1534/g3.112.005165 (PMC3618346; doi:10.1534/g3.112.005165)
Supplement: Supporting Information [file supp_3_4_585__index.html]

Assembly of the Synaptonemal Complex Is a Highly Temperature-Sensitive Process That Is Supported by PGL-1 During Caenorhabditis elegans Meiosis — Supporting Information 

# Assembly of the Synaptonemal Complex Is a Highly Temperature-Sensitive Process That Is Supported by PGL-1 During *Caenorhabditis elegans* Meiosis

## Supporting Information for Bilgir *et al.*, 2013

**Files in this Data Supplement:**

- Supporting Information - Figures S1-S5 (PDF, 669 KB)
- Figure S1 - Snip-SNP mapping of the locus of *meIs4* (containing the *lacO* array) in AV221 (PDF, 139 KB)
- Figure S2 - Karyotype analysis of AV221 using chromosome paints (PDF, 311 KB)
- Figure S3 - Decrease in homologous pairing frequency in a wild-type worm cultured at 26C (PDF, 188 KB)
- Figure S4 - Percentage of gonads classified by the substages exhibiting SYP-1 aggregates (PDF, 153 KB)
- Figure S5 - SYP-1 aggregates disappear at pachytene exit (PDF, 133 KB)
