# Supplementary material for: Assembly of the Synaptonemal Complex Is a Highly Temperature-Sensitive Process That Is Supported by PGL-1 During Caenorhabditis elegans Meiosis
Source: G3 (Bethesda). 2013 Apr 1;3(4):585–95. doi: 10.1534/g3.112.005165 (PMC3618346; doi:10.1534/g3.112.005165)
Supplement: Supporting Information [file supp_g3.112.005165_005165SI.pdf]

G3 · Genes | Genomes | Genetics

Assembly of the synaptonemal complex is a highly temperature-sensitive process that is supported by PGL-1 during *C. elegans* meiosis

Ceyda Bilgir[*1], Carolyn R. Dombecki[*], Peter F. Chen[*], Anne M. Villeneuve[**] and Kentaro Nabeshima[*]

[*] Department of Cell and Developmental Biology, University of Michigan Medical School, Ann Arbor MI 48109-2200, USA. [**] Departments of Developmental Biology and Genetics, Stanford University, School of Medicine, Stanford, CA 94305-5329, USA.

[1] Present address: Department of Molecular & Integrative Physiology, University of Michigan Medical School, Ann Arbor MI 48109-2200, USA.

Author for Correspondence: Kentaro Nabeshima
Contact:
Mailing address: 109 Zina Pithcer Pl. BSRB 3029, Ann Arbor, MI 48105, USA
Email: knabe@umich.edu
Phone: 734-615-2620
Fax: 734-615-8500

DOI: 10.1534/g3.112.005165

A

Position	*Ch. I* central	*Ch. I I* central	*Ch. III* central	*Ch. IV* central	*Ch. V* central	*X Ch.* central
Marker	K04F10	T13C2	F10E9	R105	F20D6	F45E1
Rol homozygotes	N2/CB	N2/CB	N2	N2	N2/CB	N2/CB
non-Rol homozygotes	N2/CB	N2/CB	CB	CB	N2/CB	N2/CB

B

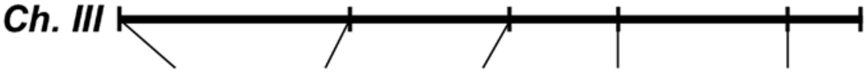

Position	-26.13	-10.04	+1.3	+8.5	+20
Marker	K02F3	H06I04	F10E9	T28D6	F54F12
Rol homozygotes	N2/CB	N2	N2	N2	N2
non-Rol homozygotes	N2/CB	CB	CB	CB	CB

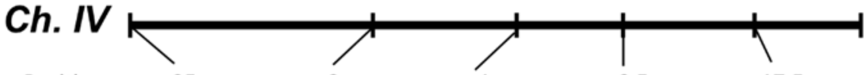

Position	-25	-8	+1	+8.5	+17.5
Marker	Y66H1A	C45G7	R105	W02A2	C35D6
Rol homozygotes	N2/CB	N2/CB	N2	N2	N2
non-Rol homozygotes	N2/CB	N2/CB	CB	CB	CB

Figure S1 Snip-SNP mapping of the locus of *meIs4* (containing the *lacO* array) in AV221. Summary of the SNP mapping analysis used to identify linkage of *meIs4* (containing the *lacO* array) to markers on both chromosomes *III* and *IV*, showing the genetic map positions of SNP markers used in this analysis; see Materials and Methods for explanation. In brief, the co-integrated marker *rol-6(su1006)* was used as a surrogate marker to track the presence of *meIs4*, which was generated in the N2 genetic background. Heterozygous F1 worms were generated by crossing the AV221 and CB4856 strains, and SNP marker genotypes were assessed both for F2 worms that were homozygous for *meIs4* (Rol homozygotes) or that lacked *meIs4* (non-Rol homozygotes). "N2/CB" indicates that both alleles were detected, reflecting independent assortment of *meIs4* and the SNP marker or crossing over between the array and the SNP marker. "N2" (in the Rol homozygotes) or "CB" (in the non-Rol homozygotes) indicates tight linkage of the SNP marker to *meIs4*. The unusual marker segregation pattern observed indicates that *meIs4* is associated with a reciprocal translocation between chromosomes *III* and *IV* (see Materials and Methods and Figure S2).

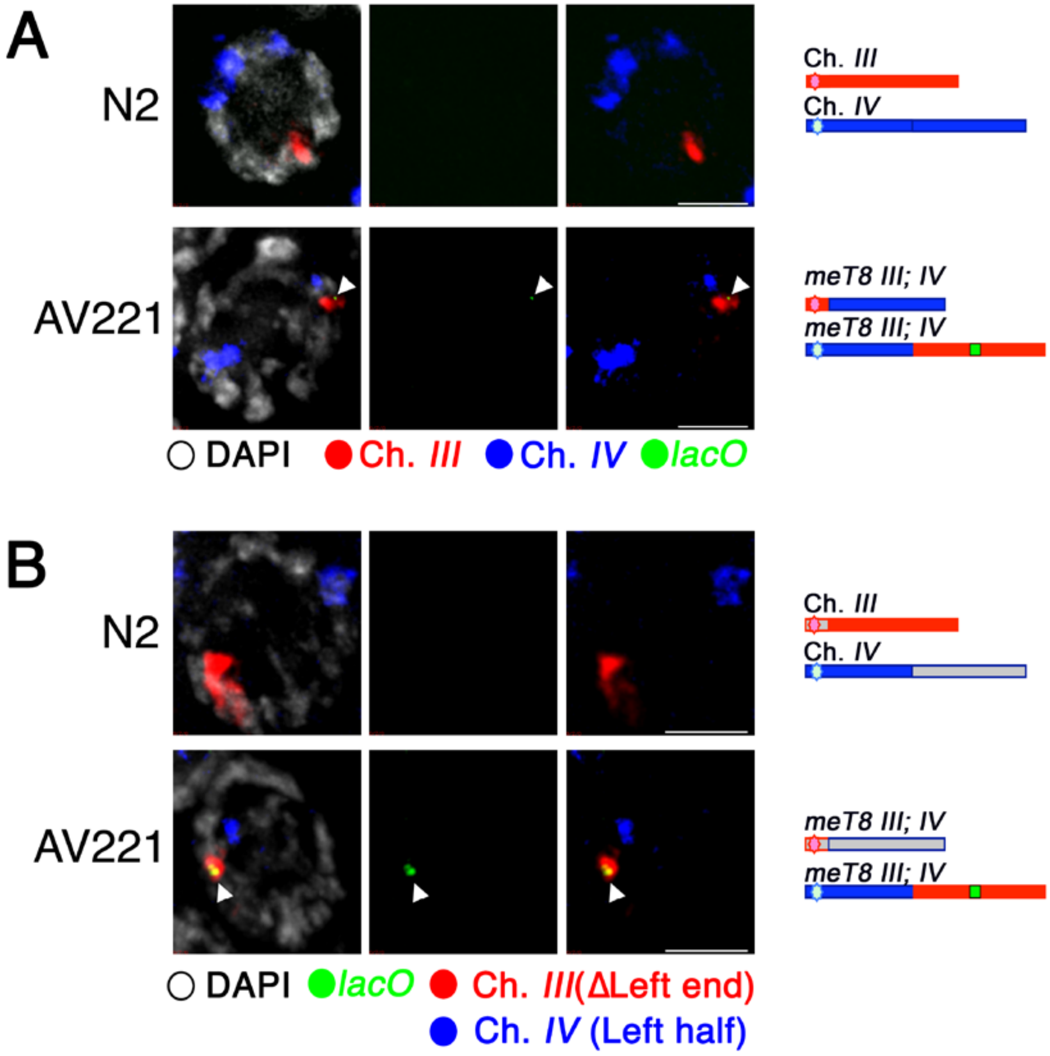

Figure S2 Karyotype analysis of AV221 using chromosome paints. (A) Projections of FISH images of a representative pachytene nucleus from the N2 wild type strain (top) and the AV221 strain (bottom), using whole-chromosome paints for chromosome *III* (red) and *IV* (blue), a FISH probe that hybridizes to the *lacO* sequence (green, arrowhead), and a DAPI counter stain (white). Schematics at the left of the images depict the karyotype of the *meT8* reciprocal translocation inferred from the combination of genetic mapping and the FISH experiments shown here; the pink and light blue symbols indicate the positions of the pairing centers. Bar: 2µm.

(B) Projections of FISH images of a representative pachytene nucleus from the N2 wild type strain (top) and the AV221 strain (bottom), using probes that hybridize to most of chromosome *III* except for a 1Mb region at the left end (red), to the left half of chromosome *IV* (blue), and to the *lacO* (green, arrowhead), and DAPI counter stain (white). Schematics at right show the portions of the karyotypes that are labeled by the probes. Bar: 2µm.

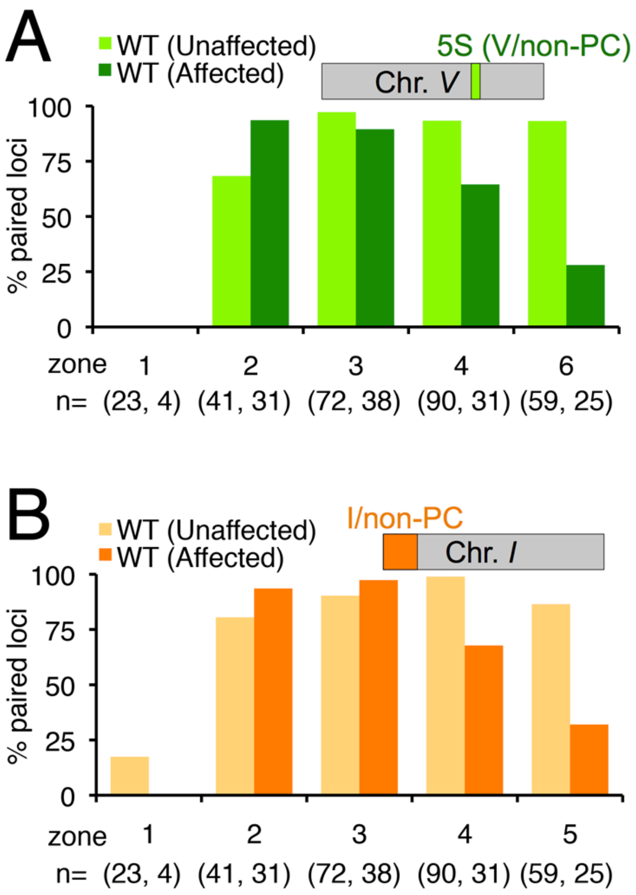

Figure S3 Decrease in homologous pairing frequency in a wild-type worm cultured at 26°C. Pairing efficiency at the indicated locus is represented as the percentage of nuclei with paired signals in each zone in the gonads of wild type worms cultured at 26°C. There are two types of phenotype: WT(Unaffected) – no decrease in pairing efficiency in pachytene stage, WT(Affected) - decrease in pairing efficiency in pachytene stage.

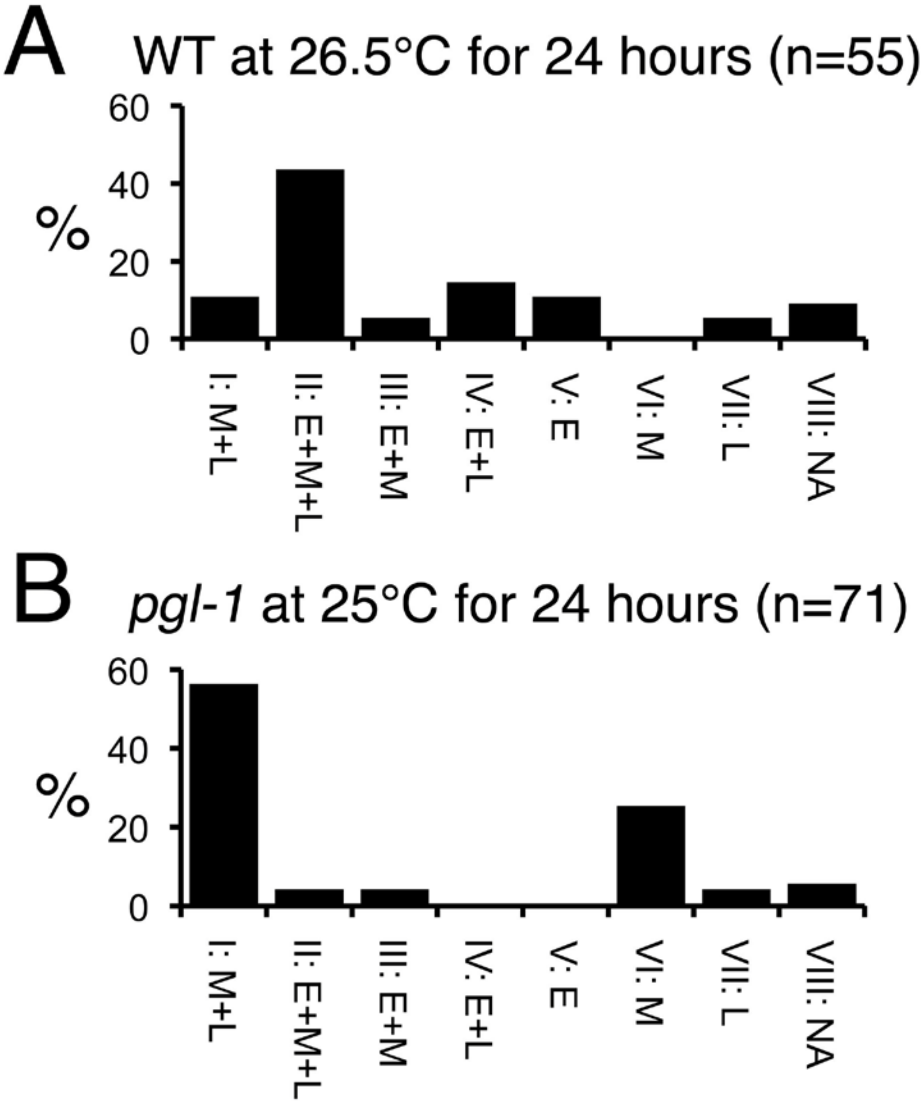

Figure S4 Percentage of gonads classified by the substages exhibiting SYP-1 aggregates. Percentage of gonads exhibiting SYP-1-aggregate formation in the indicated substages. A: wild type worms cultured at 20°C until late L4 stage and then at 26.5°C for 24 hours. B: *pgl-1(bn102)* worms cultured at 20°C until late L4 stage and then at 25°C for 24 hours. The part of each gonad corresponding to the meiotic prophase region was subdivided into three sub-zones of equal lengths; E: early meiotic prophase zone, M, middle meiotic prophase zone, L: late meiotic prophase zone. Gonads were classified by the zones that exhibit SYP-1 aggregates. Type I: M+L; Nuclei with SYP-1 aggregates are observed in both M and L, Type II: E+M+L; Nuclei with SYP-1 aggregates are observed in all three zones, Type III; Nuclei with SYP-1 aggregates are observed in both E and M, Type IV: E+L; Nuclei with SYP-1 aggregates are observed in E and L, but not in M, Type V: Nuclei with SYP-1 aggregates are observed only in E, Type VI: Nuclei with SYP-1 aggregates are observed only in M, Type VII: Nuclei with SYP-1 aggregates are observed only in L, and Type VIII: NA; Nuclei with SYP-1 aggregates are not observed.

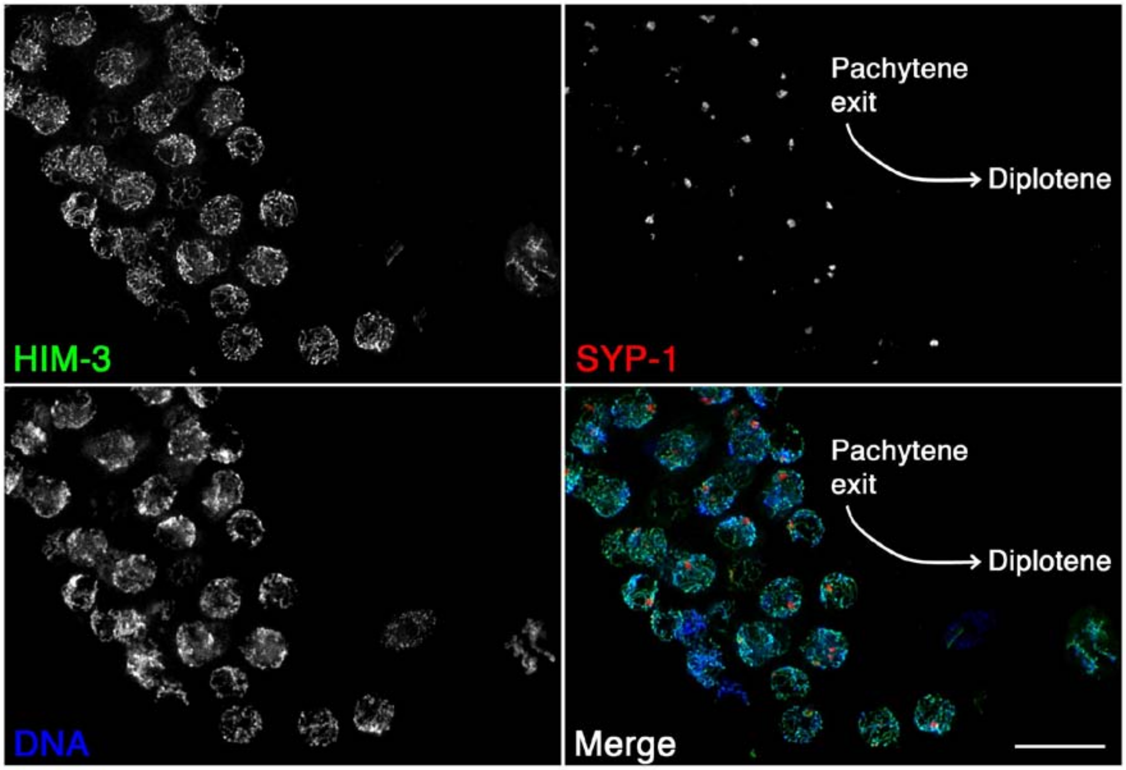

Figure S5 SYP-1 aggregates disappear at pachytene exit. A projection image of pachytene exit in the wild type cultured at 26.5°C for 24 hours stained by HIM-3/SYP-1 IF. SYP-1 aggregates become smaller and fragmented at pachytene exit and disappear in diplotene nuclei. Bar: 10µm.
