# Supplementary material for: Assembly of the Synaptonemal Complex Is a Highly Temperature-Sensitive Process That Is Supported by PGL-1 During Caenorhabditis elegans Meiosis
Source: G3 (Bethesda). 2013 Apr 1;3(4):585–95. doi: 10.1534/g3.112.005165 (PMC3618346; doi:10.1534/g3.112.005165)
Supplement: Supporting Information [file supp_g3.112.005165_FigureS1.pdf]

A

Position	*Ch. I* central	*Ch. I I* central	*Ch. III* central	*Ch. IV* central	*Ch. V* central	*X Ch.* central
Marker	K04F10	T13C2	F10E9	R105	F20D6	F45E1
Rol homozygotes	N2/CB	N2/CB	N2	N2	N2/CB	N2/CB
non-Rol homozygotes	N2/CB	N2/CB	CB	CB	N2/CB	N2/CB

B

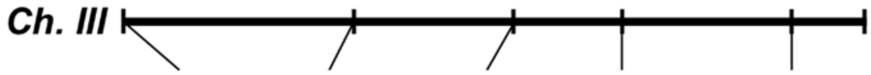

Position	-26.13	-10.04	+1.3	+8.5	+20
Marker	K02F3	H06I04	F10E9	T28D6	F54F12
Rol homozygotes	N2/CB	N2	N2	N2	N2
non-Rol homozygotes	N2/CB	CB	CB	CB	CB

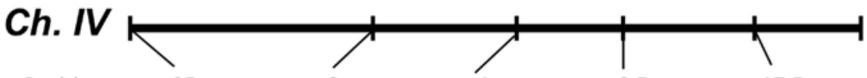

Position	-25	-8	+1	+8.5	+17.5
Marker	Y66H1A	C45G7	R105	W02A2	C35D6
Rol homozygotes	N2/CB	N2/CB	N2	N2	N2
non-Rol homozygotes	N2/CB	N2/CB	CB	CB	CB

Figure S1 Snip-SNP mapping of the locus of *meIs4* (containing the *lacO* array) in AV221. Summary of the SNP mapping analysis used to identify linkage of *meIs4* (containing the *lacO* array) to markers on both chromosomes *III* and *IV*, showing the genetic map positions of SNP markers used in this analysis; see Materials and Methods for explanation. In brief, the co-integrated marker *rol-6(su1006)* was used as a surrogate marker to track the presence of *meIs4*, which was generated in the N2 genetic background. Heterozygous F1 worms were generated by crossing the AV221 and CB4856 strains, and SNP marker genotypes were assessed both for F2 worms that were homozygous for *meIs4* (Rol homozygotes) or that lacked *meIs4* (non-Rol homozygotes). "N2/CB" indicates that both alleles were detected, reflecting independent assortment of *meIs4* and the SNP marker or crossing over between the array and the SNP marker. "N2" (in the Rol homozygotes) or "CB" (in the non-Rol homozygotes) indicates tight linkage of the SNP marker to *meIs4*. The unusual marker segregation pattern observed indicates that *meIs4* is associated with a reciprocal translocation between chromosomes *III* and *IV* (see Materials and Methods and Figure S2).
