# Supplementary material for: Assembly of the Synaptonemal Complex Is a Highly Temperature-Sensitive Process That Is Supported by PGL-1 During Caenorhabditis elegans Meiosis
Source: G3 (Bethesda). 2013 Apr 1;3(4):585–95. doi: 10.1534/g3.112.005165 (PMC3618346; doi:10.1534/g3.112.005165)
Supplement: Supporting Information [file supp_g3.112.005165_FigureS2.pdf]

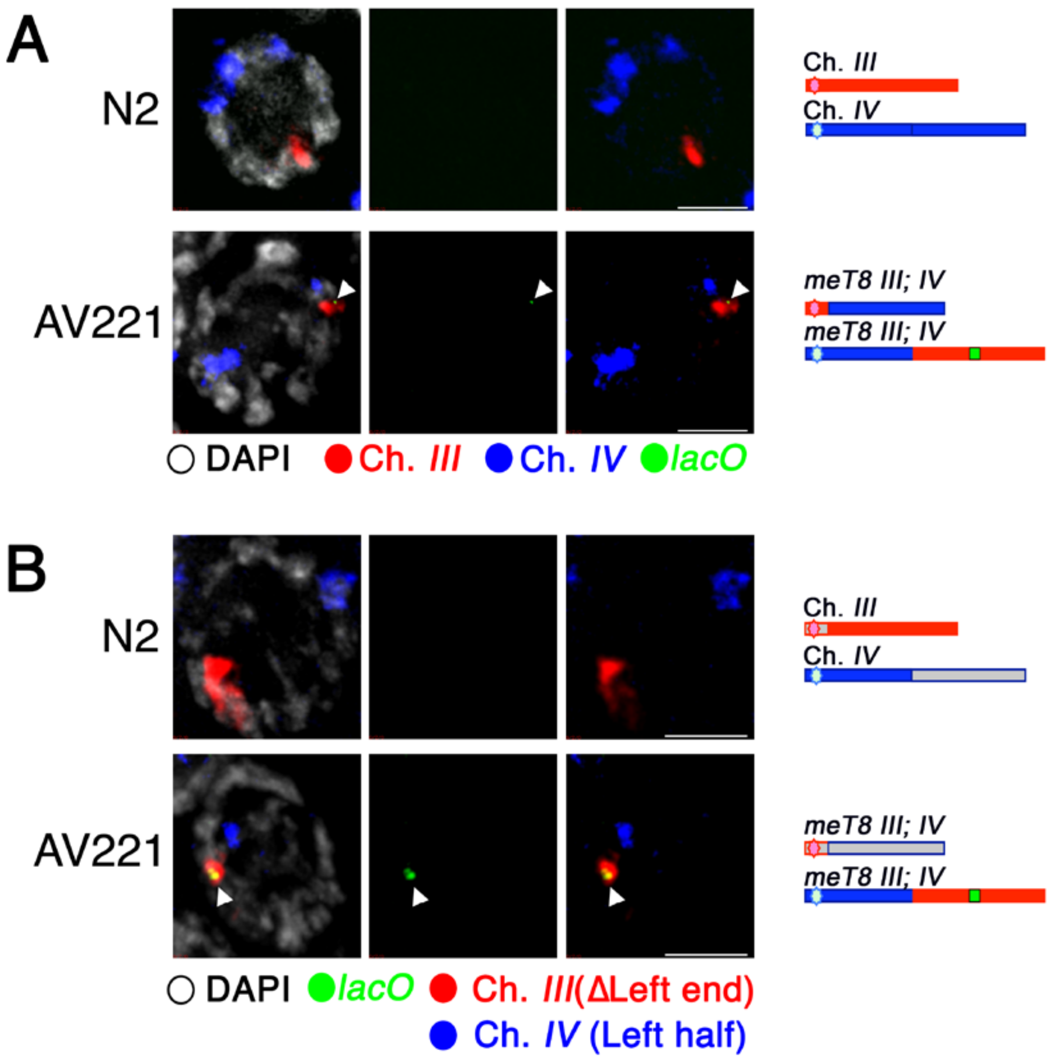

Figure S2 Karyotype analysis of AV221 using chromosome paints. (A) Projections of FISH images of a representative pachytene nucleus from the N2 wild type strain (top) and the AV221 strain (bottom), using whole-chromosome paints for chromosome *III* (red) and *IV* (blue), a FISH probe that hybridizes to the *lacO* sequence (green, arrowhead), and a DAPI counter stain (white). Schematics at the left of the images depict the karyotype of the *meT8* reciprocal translocation inferred from the combination of genetic mapping and the FISH experiments shown here; the pink and light blue symbols indicate the positions of the pairing centers. Bar: 2µm.

(B) Projections of FISH images of a representative pachytene nucleus from the N2 wild type strain (top) and the AV221 strain (bottom), using probes that hybridize to most of chromosome *III* except for a 1Mb region at the left end (red), to the left half of chromosome *IV* (blue), and to the *lacO* (green, arrowhead), and DAPI counter stain (white). Schematics at right show the portions of the karyotypes that are labeled by the probes. Bar: 2µm.
