# Supplementary material for: Assembly of the Synaptonemal Complex Is a Highly Temperature-Sensitive Process That Is Supported by PGL-1 During Caenorhabditis elegans Meiosis
Source: G3 (Bethesda). 2013 Apr 1;3(4):585–95. doi: 10.1534/g3.112.005165 (PMC3618346; doi:10.1534/g3.112.005165)
Supplement: Supporting Information [file supp_g3.112.005165_FigureS3.pdf]

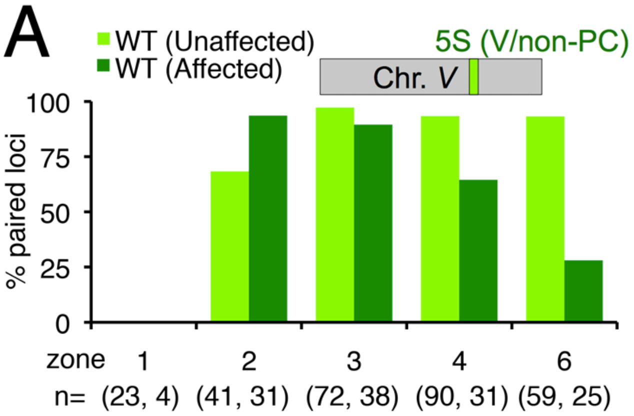

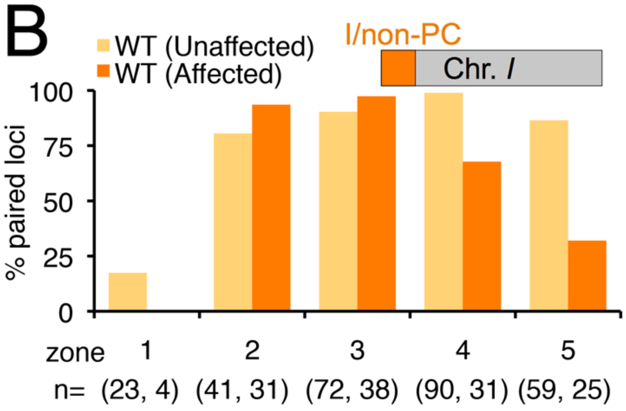

Figure S3 Decrease in homologous pairing frequency in a wild-type worm cultured at 26°C. Pairing efficiency at the indicated locus is represented as the percentage of nuclei with paired signals in each zone in the gonads of wild type worms cultured at 26°C. There are two types of phenotype: WT(Unaffected) – no decrease in pairing efficiency in pachytene stage, WT(Affected) - decrease in pairing efficiency in pachytene stage.
