# Supplementary material for: Assembly of the Synaptonemal Complex Is a Highly Temperature-Sensitive Process That Is Supported by PGL-1 During Caenorhabditis elegans Meiosis
Source: G3 (Bethesda). 2013 Apr 1;3(4):585–95. doi: 10.1534/g3.112.005165 (PMC3618346; doi:10.1534/g3.112.005165)
Supplement: Supporting Information [file supp_g3.112.005165_FigureS4.pdf]

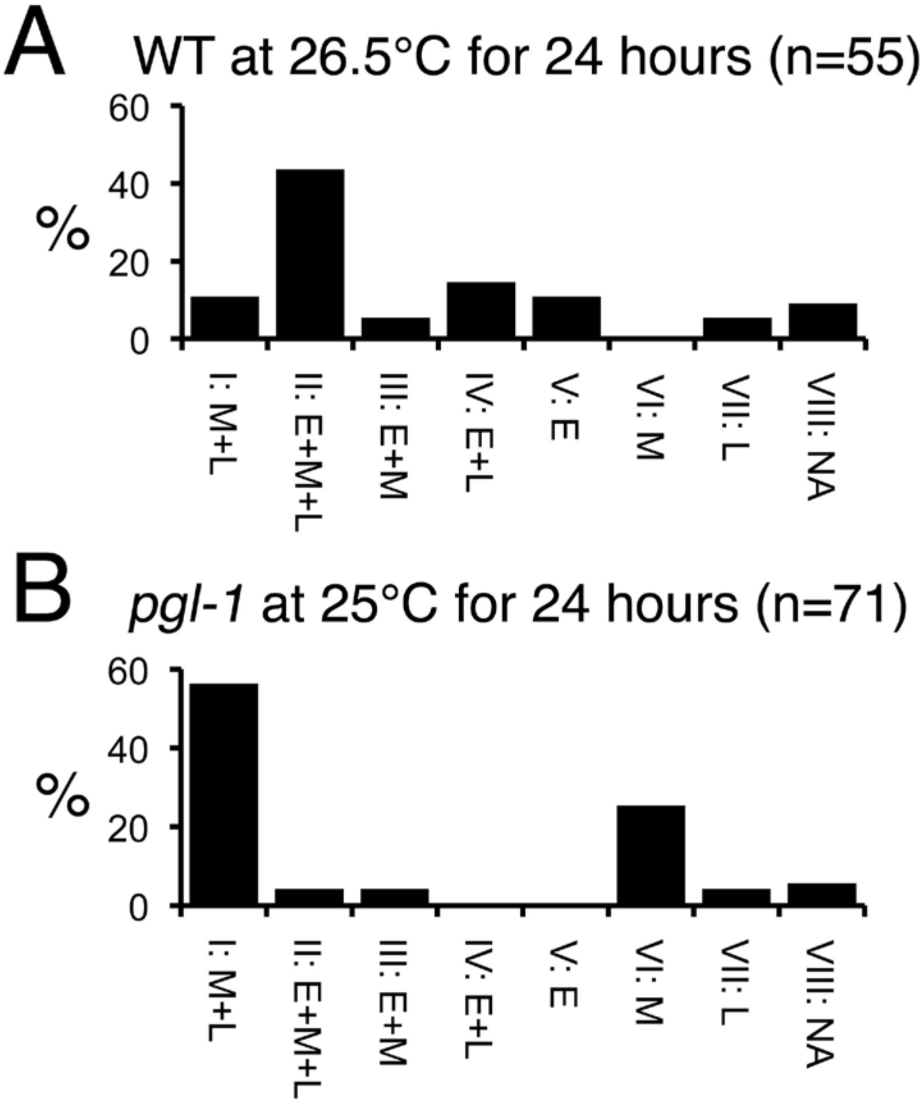

Figure S4 Percentage of gonads classified by the substages exhibiting SYP-1 aggregates. Percentage of gonads exhibiting SYP-1-aggregate formation in the indicated substages. A: wild type worms cultured at 20°C until late L4 stage and then at 26.5°C for 24 hours. B: *pgl-1(bn102)* worms cultured at 20°C until late L4 stage and then at 25°C for 24 hours. The part of each gonad corresponding to the meiotic prophase region was subdivided into three sub-zones of equal lengths; E: early meiotic prophase zone, M, middle meiotic prophase zone, L: late meiotic prophase zone. Gonads were classified by the zones that exhibit SYP-1 aggregates. Type I: M+L; Nuclei with SYP-1 aggregates are observed in both M and L, Type II: E+M+L; Nuclei with SYP-1 aggregates are observed in all three zones, Type III; Nuclei with SYP-1 aggregates are observed in both E and M, Type IV: E+L; Nuclei with SYP-1 aggregates are observed in E and L, but not in M, Type V: Nuclei with SYP-1 aggregates are observed only in E, Type VI: Nuclei with SYP-1 aggregates are observed only in M, Type VII: Nuclei with SYP-1 aggregates are observed only in L, and Type VIII: NA; Nuclei with SYP-1 aggregates are not observed.
