# Supplementary material for: Assembly of the Synaptonemal Complex Is a Highly Temperature-Sensitive Process That Is Supported by PGL-1 During Caenorhabditis elegans Meiosis
Source: G3 (Bethesda). 2013 Apr 1;3(4):585–95. doi: 10.1534/g3.112.005165 (PMC3618346; doi:10.1534/g3.112.005165)
Supplement: Supporting Information [file supp_g3.112.005165_FigureS5.pdf]

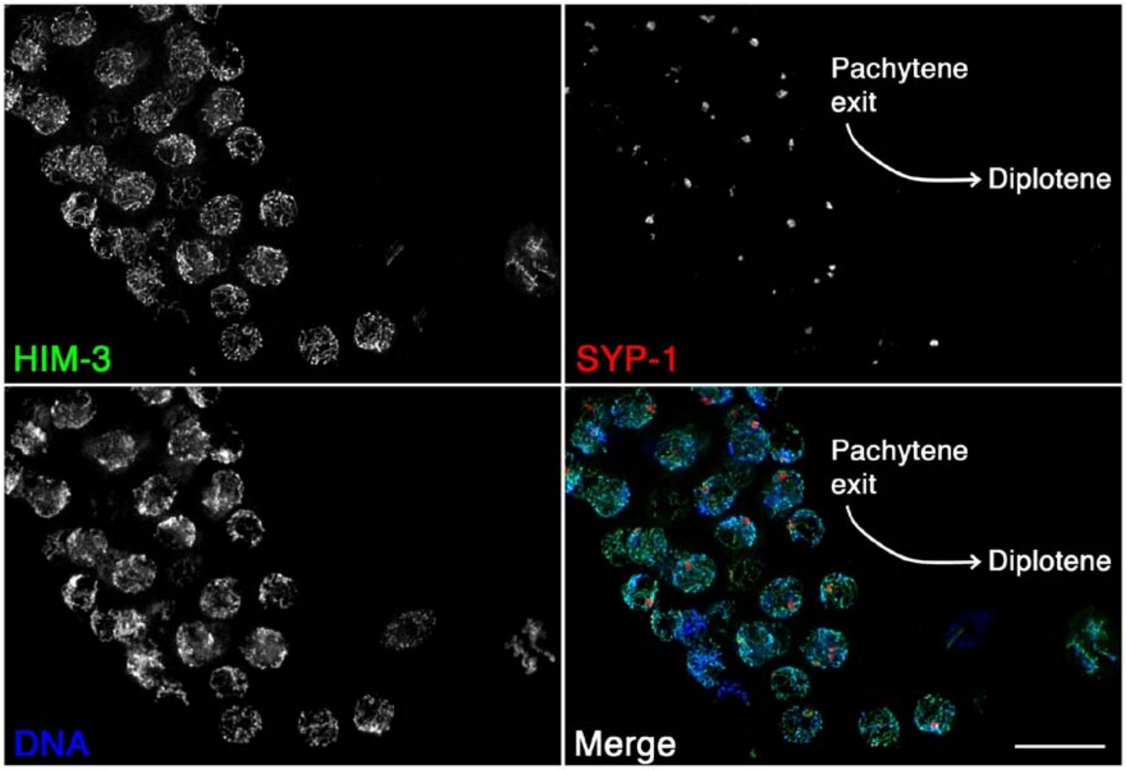

Figure S5 SYP-1 aggregates disappear at pachytene exit. A projection image of pachytene exit in the wild type cultured at 26.5°C for 24 hours stained by HIM-3/SYP-1 IF. SYP-1 aggregates become smaller and fragmented at pachytene exit and disappear in diplotene nuclei. Bar: 10µm.
